# Potential effectiveness of traditional Chinese medicine for cardiac syndrome X (CSX): a systematic review and meta-analysis

**DOI:** 10.1186/1472-6882-13-62

**Published:** 2013-03-17

**Authors:** Jia-ying Wang, Lu Xiao, Jing Chen, Jing-bo Zhai, Wei Mu, Jing-yuan Mao, Hongcai Shang

**Affiliations:** 1 The Center of Evidence Based Medicine, Tianjin University of Traditional Chinese Medicine, Anshan West Road, Tianjin 300193, China; 2 Tianjin Institution of Clinical Evaluation, Tianjin University of Traditional Chinese Medicine, Yuquan Road, Tianjin 300193, China; 3 Cardiovascular Department, The First Teaching Hospital of Tianjin University of Traditional Chinese Medicine, Anshan West Road, Tianjin 300193, China

## Abstract

**Background:**

Treatment of cardiac syndrome X with unknown pathological mechanism remains a big challenge for clinicians. Complementary and alternative medicine may bring a new choice for its management. The aim of this study is to evaluate the clinical effects of traditional Chinese medicine on cardiac syndrome X patients.

**Methods:**

We systematically searched databases such as Cochrane CENTRAL, PubMed, EMBASE, CBM, Chinese National Knowledge Infrastructure (CNKI), WanFang and VIP, and handsearched relevant journals to identify randomized controlled trials. Following the steps of systematic review recommended by the Cochrane group, we assessed the quality of included studies, extracted valid data and undertook meta-analysis.

**Results:**

Twenty one moderate-to low-quality randomized controlled trials involving 1143 patients were included. The results showed that traditional Chinese medicine could improve angina [OR=1.34, 95% CI: 1.2 to 1.50], electrocardiogram (ECG), endothelin-1 (ET-1) levels, prolong exercise duration in treadmill tests, and reduce angina frequency per week compared with routine treatment. No other side effect was reported except two cases of stomach pain.

**Conclusion:**

Compared with conventional treatment, traditional Chinese medicine shows the potential of optimizing symptomatic outcomes and improving ECG and exercise duration. The efficacy of TCM may find explanation in its pharmacological activity of adjusting the endothelial function. TCM, as a kind of alternative and complementary medicine, may provide another choice for CSX patients.

## Background

Cardiac syndrome X (CSX, also called microvascular angina) refers to typical stable angina that is exclusively or predominantly induced by effort. Upon diagnostic investigation, findings are comparable to those of myocardial ischemia, showing normal or near-normal coronary arteries on angiography and an absence of any other specific cardiac diseases (e.g., variant angina, cardiomyopathy, or valvular diseases) [1]. Approximately 10% to 20% of affected patients suspected to have angina reportedly turned out to have negative coronary angiogram results [2]. Although the long-term prognosis of CSX does not necessarily include increased mortality, patients’ quality of life is invariably affected and the incidence rates of cardiovascular and cerebrovascular events are increased [3]. Exacerbated and recurrent angina may also lead to physical discomfort, frequent hospital readmissions, or even repeat coronary angiography, imposing patients with a huge economic burden [4]. Moreover, patients with CSX tend to show high scores on psychological inventories that measure anxiety and depression [5].

The etiology and pathology of CSX remain unclear. Although hypotheses claiming that the disease is associated with endothelial dysfunction, inflammation, oxidative stress, or estrogen deficiency have been formulated, they lack evidence [6,7]. Internationally, conventional drugs against ischemia, such as beta-blockers, calcium antagonists, xanthine derivatives, angiotensin-converting enzyme inhibitors, estrogen, and statins, are recommended for clinical use in patients with CSX. However, their respective curative effects remain controversial [8-10]. We believe that alternative and complementary medicine may provide additional treatment options. As more papers on treatment with Traditional Chinese Medicine (TCM) were published in recent years, it was suggested that Chinese herbs might bring health benefits for patients with CSX [3]. TCM has a unique system of interpreting the etiology and pathology of CSX [11], allowing this syndrome to be treated accordingly. Moreover, modern pharmacological studies have found that the active ingredients in many of these herbs have functions related to endothelial protection, anti-inflammation, antioxidative stress, and improvement of estrogen function, all of which are believed to be effective in the pathology of CSX [12-17]. Therefore, the relatively inexpensive TCM treatment for CSX is worthy of attention. This study aimed to systematically and objectively evaluate the clinical curative effect and safety of TCM for CSX based on a general understanding of previous research on CSX.

## Methods

This systematic review is conducted according to the Preferred Reporting Items for Systematic Reviews and Meta- analyses: Additional file 1: The PRISMA Statement.

### Ethics

Data for this study was acquired through previously published work, no patient or hospital data was accessed. Therefore, written consent and institutional ethical review was not required for this research.

### Search strategy

We systematically searched for studies on CSX published in either Chinese or English. The databases that were searched included the Cochrane Central Register of Controlled Trials (Issue 3, 2011), PubMed (1978–2011.9), EMBASE (1995–2011), MEDLINE (1984–2010.5), CINAL (1984–2010.5), CNKI (1980–2011), CBM disc (1981–2011.9), WANFANF (1980–2011.9), and VIP (1989–2011.9). The search strategy was formulated using MeSH terms in combination with free words. Details of the search strategy for the English databases are as follows:

1. Angina, Microvascular

2. Cardiac Syndrome X

3. X Syndrome, Angina

4. Angina X Syndrome

5. Angina X Syndromes

6. Syndrome, Angina X

7. Syndrome X, Cardiac

8. Angina Pectoris with Normal Coronary Arteriogram

9. Syndrome X, Angina

10. Angina Syndrome X

11. Angina Syndrome Xs

12. Syndrome Xs, Angina

13. Chinese medicine [All Fields]

14. (1 or 2 or 3 or 4 or 5 or 6 or 7 or 8 or 9 or 10 or 11 or 12) [Title/Abstract]

15. 13 AND 14

Details of the search strategy for the Chinese databases are as follows:

The Chinese characters used to perform the search are hereafter stated in Chinese ***pinyin***

For example:

(Zhuti (title/abstract) = *xinzangXzonghezheng* (cardiac syndrome  X)  or  *weixueguanxingxinjiaotong*  (microvasculature)

We also checked the references of literature for possible identification of relevant studies. Electronically inaccessible journal articles were manually retrieved.

### Inclusion criteria

•The study is a randomized controlled trial (RCT).

•The study includes participants diagnosed with CSX by the criteria listed in *ANGINA PECTORIS AND NORMAL CORONARY ARTERIES: CARDIAC SYNDROME X*[18].

•The study includes intervention and comparison of any of the following: 

A. TCM + routine treatment* vs. routine treatment

B. TCM + routine treatment vs. routine treatment + placebo.

C. TCM vs. routine treatment

•*Routine treatment includes nitrate medications, beta-blockers, angiotensin-converting enzyme inhibitors, angiotensin receptor blockers, and calcium channel blockers, as recommended in the essay *Therapeutic Options for the Management of Patients with Cardiac Syndrome X*[19].

•Outcomes to be measured include symptom improvement and electrocardiograph (ECG) changes and/or treadmill test results. Any adverse event defined in “ICH—GCP 1997” [20] should be recorded.

### Exclusion criteria

We excluded studies with unclear diagnostic criteria and without full texts or the use of other TCM therapeutic methods except herbs. Intervention for the treatment group was limited to Chinese herbs. Combinations of herbs and other forms of treatment (e.g., acupuncture or moxibustion) were excluded.

### Study selection

Two researchers (W.J.Y. and X.L.) independently undertook the aforementioned search and managed the search results using Note Express 2 software. Ineligible studies were excluded after reading the title and abstract, and the full texts of the remaining studies were scanned for confirmation. When disagreement arose between the two researchers, they sought help from a third reviewer (S.H.C.).

### Data collection

Both researchers (W.J.Y. and X.L.) independently extracted data from all included studies and input them into two Excel sheets. One sheet contained the general information of the studies, including the authors’ names (the first author was listed if there was more than one), publication date, sample size, age of participants, gender, interventions and comparisons, treatment courses, and outcomes. The other sheet included items for quality assessment listed in the Cochrane risk of bias tool [21]. The above information was then carefully checked by two other researchers (C.J. and Z.S.). Any disagreement was resolved through discussion, and errors were corrected.

### Statistical analysis

The two researchers (W.J.Y. and X.L.) used Review Manager 5.0.2, provided by the Cochrane corporative network, to analyze the data. Effect measures were presented using odds ratios (OR) for dichotomous data and weighted mean differences (WMD) or standardized mean differences (SMD) for continuous data, both with 95% confidence intervals (CI). The chi-square test was used to test heterogeneity across studies [22] with a significance level of 0.05. Data were analyzed with a fixed-effect model if no statistical heterogeneity was observed (P > 0.05 or I^2^ < 50%). In the presence of heterogeneity, the two researchers (W.J.Y. and X.L.) checked the data entered and explored the variation by conducting subgroup analysis. If the variation could not be explained, we performed a random-effects meta-analysis and interpreted the effect measure with care. A funnel plot was formed to detect publication bias [23].

### Subgroup analysis, sensitivity, and meta-regression

Subgroup analysis was conducted to show the magnitudes of the effects in different subgroups and determine whether there was a different effect of an intervention in different situations. Meta-regression analysis was conducted to identify possible causes for differences across the groups.

## Results

**Figure 1:**
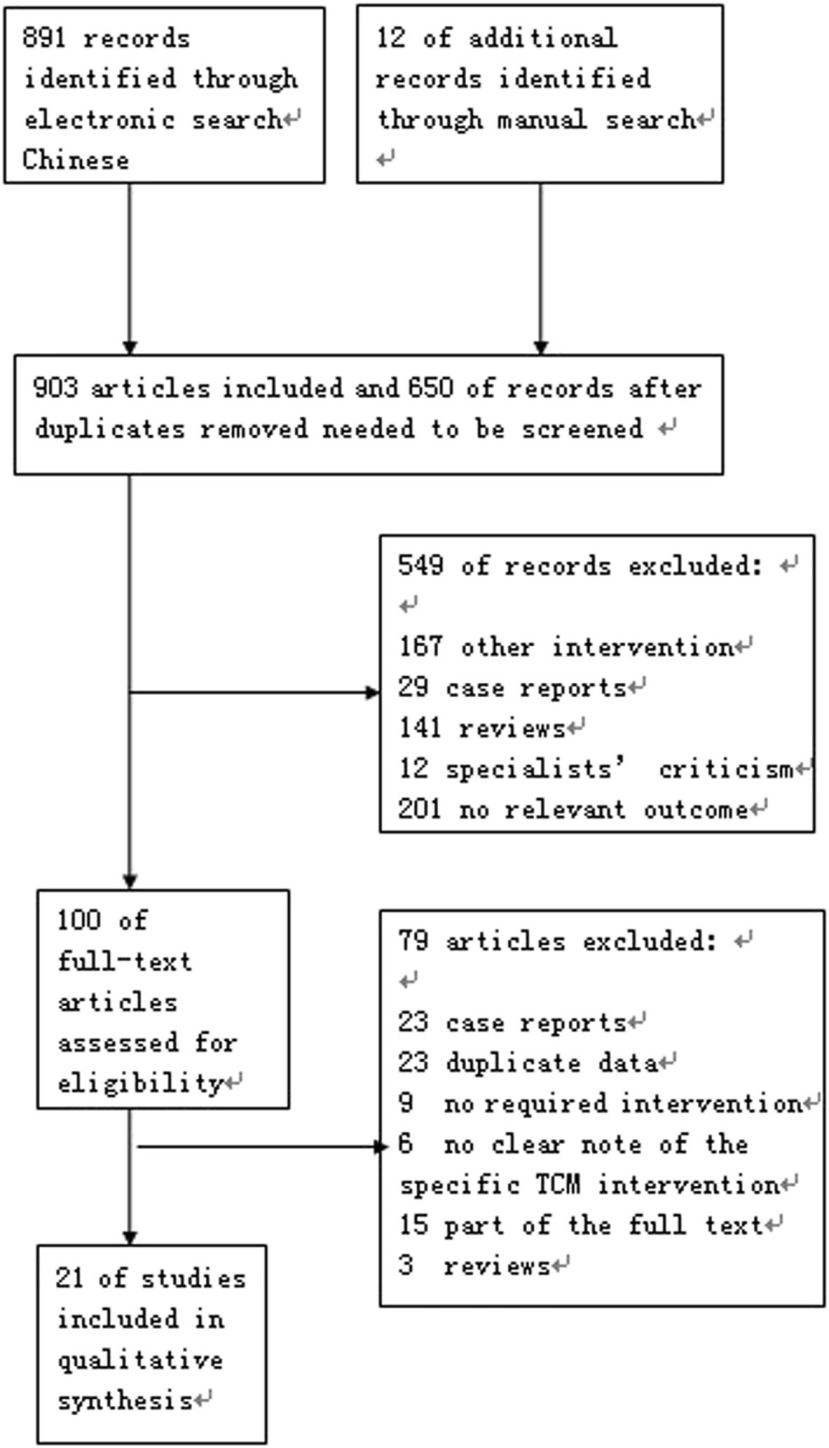
A flow diagram of study selection followed the PRISMA template.

A total of 903 studies (900 in Chinese and 3 in English) were identified through electronic searches; 101 of them were included for further assessment after both researchers examined the title and abstract of all studies and excluded duplicates, animal experiments, reviews, and others. We read the full texts of all 101 articles and discovered and excluded other ineligible studies. The final result included 21 articles [24-44], all in Chinese (see Figure 1).

### General characteristics of the included studies

All 21 RCTs, which involved 1143 patients aged 35 to 76 years, were published between 2002 and 2011. In all studies excluding two (Liu 2008 and Zhu 2003), female participants outnumbered male participants. Treatment courses ranged from 7 days to 1 year. In one study (Xv 2002), the duration was 7 days, and in another study (Wang 2009), it was 14 days. The treatment length was 15 days in three studies^34,36,42^ and 28 days in nine studies. The longest treatment length (Lu 2007) was 84 days. The other two studies (Liu 2008 and Zhu 2003) did not report the medication treatment course. Only two studies (Bi 2003 and Xv 2002) reported follow-up data.

Of the 21 RCTs, 12 compared TCM plus routine treatment with routine treatment alone, eight compared TCM with routine treatment, and the remaining RCT (Liang 2005) compared TCM combined with routine treatment versus placebo, which were combined with routine treatment. TCM interventions included Chinese patented drugs (12 studies), decoction (seven studies), and injection (two studies). With regard to outcome measures, angina improvement was reported in 15 studies, angina frequency in nine studies, and treadmill test results in 11 studies. Eight trials used ECG improvement as the primary outcome measure, two trials used TCM syndrome improvement, and seven used changes in cytokine levels such as endostatin ET-1 or C-reactive protein (CRP). Only one study reported fractional flow reserve. Ten studies reported safety outcomes (see Table 1).

**Table 1:** General characteristic of the included studies

**Studies**	**Case**	**Age**	**Gender**	**Co**	**F**	**CP**	**Outcome**	**AE**	**CI**	**Type of TI**	**TI**
Year*	**(T/C)**		**(M/F)**	**(d)**							
Sun 2007 [24]	64(34/30)	37~62	T:13/21C:11/19	56	0	y	AF,TT	U	RT	Chinese patent drug	*Shenwuguanxinning* granules 15 g tid + RT
Ge 2011 [25]	39(19/20)	45~56	10/29	28	0	y	A,AF,TTET-1	U	RT	Decoction	Nourishing Qi and Blood Decoction ^1^ 150 mL qd + RT
Peng 2011 [26]	46(23/23)	35~65	18/28	56	0	y	A,AF,TT	N	RT	Chinese patent drug	*Xinnaotong* capsule 0.8 g bid + RT
Niu 2008 [27]	50(30/20)	35~65	21/29	28	0	y	A,AF,ECG,TT,TCM	N	RT	Chinese patent drug	*Xinxuean* granules 15 g tid + RT
Gao 2005 [28]	63(33/30)	45~64	0/63	28	0	y	A	U	RT	Decoction	*Xiaoyao* decoction^2^ 150 mL bid
Xu 2011 [29]	30(15/15)	35~60	10/20	28	0	y	A,AF,TT	U	RT	Chinese patent drug	*Wenxin* granules^3^ 9 g, tid + RT
Bi 2003 [30]	60(30/30)	44~61	27/33	28	6 m	y	A	N	RT	Chinese patent drug	*Tongxinluo* 1.52 g, tid
Wang 2009 [31]	55(28/27)	43~65	T: 5/23 C: 3/24	14	0	y	A,ECG,TCM,TTET-1,NOhs-CRP	N	RT	Decoction	*Kuanxionghuoxue* decoction^4^ 150 ml bid + RT
Xu LJ 2002 [32]	56(36/20)	31~48	T:23/13 C:13/7	7	7d	y	A,ET-1,Holter	U	RT	Chinese patent drug	*Quanshi* capsule 1.5 g tid
Liu 2008 [33]	58(38/20)	36~55	37/21	U	0	y	A,ECG	U	RT	Chinese patent drug	*Naoxintong* tablets, 1.6 g, tid
Zhang 2002 [34]	40(20/20)	30~59	18/22	15	0	y	A,ECG	U	RT	Decoction	*Taohongsiwu* decoction^5^ 150 mL qd
Lu 2007 [35]	114(72/72	18~60	56/88	84	0	y	TT,ECG	N	RT	Chinese patent drug	*Tongxinluo* capsule 1.52 g tid
Zhu 2003 [36]	60(30/30)	30~59	38/22	15	0	y	A,ECG	U	RT	Decoction	*Xuefuzhuyu* decoction ^6^150 mL qd
Liang 2005 [37]	42(21/21)	51~60	12/30	28	0	y	A,AF, TCM,TT hs-CRP	N	RT placebo	Chinese patent drug	*Danshen* tablets, three tablets per time, tid + RT
Yuan 2008 [38]	40(20/20)	48~67	T:9/11 C:13/7	28	0	y	AF,TT	U	RT	Injection	*Danhong* injection 30 ml + 5% glu or 0.9% NaCI 250 ml, iv, qd
Wang GF 2008 [39]	36(18/18)	33~70	T:3/15 C:2/16	28	0	y	AF,TT,CRP,ET-1,NO	N	RT	Decoction	*Guanmailing* decoction^7^ 150 ml bid+ RT
Zhang SL 2008 [40]	72(36/36)	37~62	T:6/30 C:3/31	20	0	y	A	N	R	Chinese patent drug	*Guanmaining*^8^ 10 ml tid + *xiaoyao* pill, 8 pills, tid+ RT
Zhang XY 2008 [41]	68(48/28)	42~58	T:11/37 C:6/14	21	0	y	A,ECG	U	RT	Decoction	Individualized Chinese formulas against different patterns of syndrome^9^ 150 ML bid+ RT
Wu 2010 [42]	50(26/24)	39~50	T:10/16 C: 9/15	15	0	y	AF,TT, CRP	N	RT	Injection	*Shuxuening* injection 20 ml+ 0.9% NaCI 250 ml, iv, qd+ RT
Li 2009 [43]	68(36/32)	49~64	T:16/20 C:14/18	30	0	y	ECG, blood lipid, ET-1,No,	Y	RT	Chinese patent drug	*Tongxinluo* capsule, 2.14 g, tid + RT
Feng 2005 [44]	32(16/16)	37~51	T:7/9 C:8/8	28	0	y	AF,TT ET-1	U	RT	Chinese patent drug	*Tongxinluo* capsule, 1.14 g tid+ RT

Table Legends :

* Studies were marked by the surname of the first author followed by the year of publication . An abbreviation of the first name was appended if authors share the same surname.

Abbreviation: *Co(d)*-medication course; *F*-follow-up; *C*-compatibility; *Y-yes*; U-unclear; *N-no*; RT-routine treatment; *A*-the symptom of angina; *AF*-angina frequency; *TT*-treadmill test; *ECG*-electrocardiogram; *AE*-adverse event; *CI*-intervention in the controlled group; *TI*- intervention in the treatment group; *T*- treatment group; *C*-controlled group, *d*-day; *m*-month.

Herbal supplements in the decoction:

1 Nourishing Qi and Blood Decoction (huangqi 30 g, chaihu 12 g, gualoupi 15 g, xiebai 15 g, houpou 15 g, jiangbanxia 9 g, zhiqiao 9 g, huanglian 3 g, tanxiang 9 g, dansen 9 g, chuanxiong 9 g, danggui 6 g, baishao 12 g, baizhu 15 g, zhigancao 6 g, dangshen 12 g).

2 *Xiaoyao* decoction (chaihu 10 g, danggui 15 g, chishao15g, baishao 15 g, gancao 6 g, danshen 30 g, chuanxiong 10 g, honghua 10 g, sanqi 3 g, qianhu 10 g).

3 *Wenxin* granules (dangshen, huangjing, sanqi, hupo, gansong).

4 *Kuanxionghuoxue* decoction(chaihu 10 g, zhiqiao 12 g, guoloupi 15 g, xiebai 10 g, fuling 12 g, baizhu 12 g, chenpi12g, chuanxiong 10 g, danggui 12 g, danshen 15 g, yanhusuo 15 g, honghua 15 g).

5 *Taohongsiwu* decoction (taoren 10 g, honghua 10 g, danggui 15 g,danshen 30 g, sanqi powder 3 g, guoloupi 18 g, gegen 30 g, chuanxiong 10 g, guizhi 6 g).

6 *Xuefuzhuyu* decoction(danggui 15 g, taoren 15 g, honghua 15 g, chishao 15 g, chuanxiong 15 g, shengdihuang 15 g, niuxi 10 g, zhiqiao 10 g, chaihu 10 g, jiegeng 6 g).

7 *Guanmailing* formula(danshen 20 g, tanxiang 10 g, sharen 10 g, gualou 10 g, xiebai 10 g, zhiqiao 10 g, sanqi 10 g, chuanxiong 10 g, yinxingye 10 g, honghua 6 g, jiuxiangchong 10 g, 150 ml one pocket).

8 *Guanmaiding* (huangqi, sanleng, xiebai).

9 Individualized Chinese herbal formula against different patterns of syndrome.

Basic formula: chaihu, zhiqiao, chishao, baishao, xiangfu, chuanxiong, fuling, chenpi, suanzaoren, danshen, yuanzhi, taoren, honghua, danggui and gancao.

Adding huangqi, dangshen and baizhu for patients with Qi-deficiency; Adding anpi and zhizi for patients with excessive heat; Adding gualou, xiebai and baikouren for patients with phlegm.

### Quality assessment of the included studies

All 21 articles mentioned the word “randomization,” but only two studies (Sun 2007 and Bi 2003) elaborated on the randomization method (random number table). Only two studies (Peng 2011 and Niu 2008) mentioned the performance of single blinding. None mentioned allocation concealment or intention-to-treat analysis (see Table 2).

**Table 2:** Assessment of the methodological quality for individual trials

Studies year*	**Randomization**	**Allocation concealment**	**Blinding**	**Loss to follow-up**	**ITT analysis**
Sun 2007 [24]	yes	unclear	unclear	no	unclear
Ge 2011 [25]	yes	unclear	unclear	no	unclear
Peng 2011 [26]	yes	unclear	yes	no	unclear
Niu 2008 [27]	yes	unclear	yes	no	unclear
Gao 2005 [28]	yes	unclear	unclear	no	unclear
Xu 2011 [29]	yes	unclear	unclear	no	unclear
Bi 2003 [30]	yes	unclear	unclear	no	unclear
Wang 2009 [31]	yes	unclear	unclear	no	unclear
Xu LJ 2002 [32]	yes	unclear	unclear	no	unclear
Liu 2008 [33]	yes	unclear	unclear	no	unclear
Zhang 2002 [34]	yes	unclear	unclear	no	unclear
Lu 2007 [35]	yes	unclear	unclear	no	unclear
Zhu 2003 [36]	yes	unclear	unclear	no	unclear
Liang 2005 [37]	yes	unclear	unclear	no	unclear
Yuan 2008[38]	yes	unclear	unclear	no	unclear
Wang GF 2008 [39]	yes	unclear	unclear	no	unclear
Zhang SL 2008 [40]	yes	unclear	unclear	no	unclear
Zhang XY 2008 [41]	yes	unclear	unclear	no	unclear
Wu 2010 [42]	yes	unclear	unclear	no	unclear
Li 2009 [43]	yes	unclear	unclear	no	unclear
Feng 2005 [44]	yes	unclear	unclear	no	unclear

### Efficacy and safety analysis

The 21 RCTs were divided into two subgroups for further analysis with consideration of clinical heterogeneity across the studies. Group A (13 studies) compared TCM plus routine treatment with routine treatment alone, while Group B (eight studies) evaluated the effects of TCM relative to routine treatment. One study that compared the effects of TCM plus routine treatment with that of placebo plus routine treatment was assigned to Group A. Descriptions and interpretations of angina and ECG improvement for patients in all studies followed the rules prescribed in the *Efficacy Criteria for Angina of Coronary Heart Disease*[45]. TCM syndromes were determined against the same criteria: *Guideline for Clinical Research of Chinese Medicine (New Drug)*[46].

### Subgroup analysis

Group A: TCM + RT vs. RT

**Figure 2:**
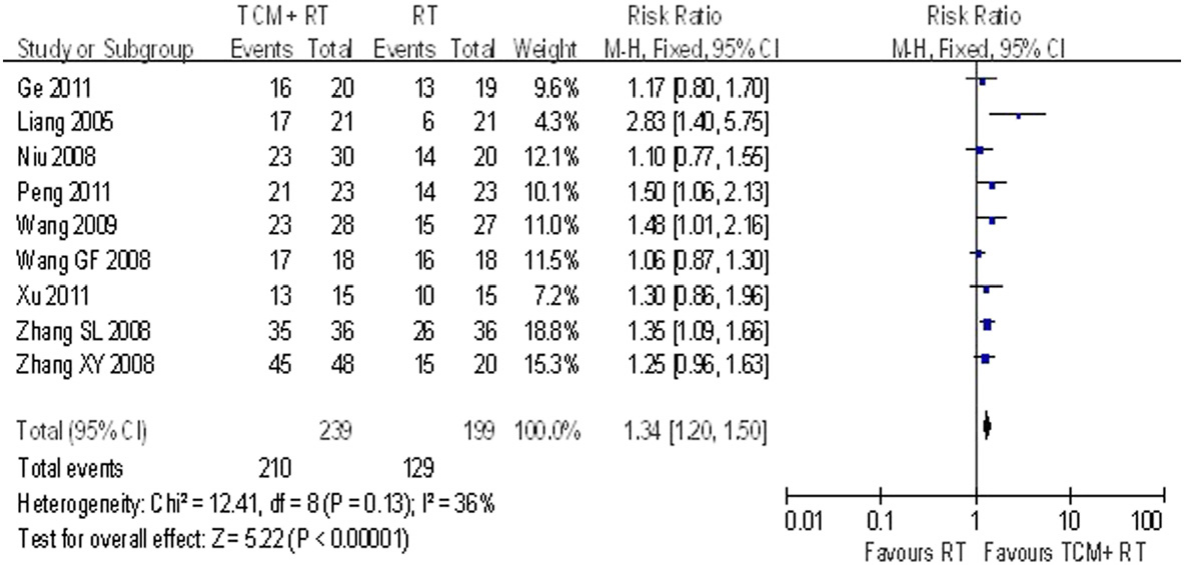
Angina improvement.

Nine studies involving 239 participants in the treatment group reported angina improvement. No statistical heterogeneity was found among these studies (P = 0.13 > 0.05, I^2^ = 36%) (see Figure 2). The results showed that TCM combined with routine treatment was more effective than routine treatment alone (OR = 1.34, 95% CI = 1.2–1.50).

**Figure 3:**
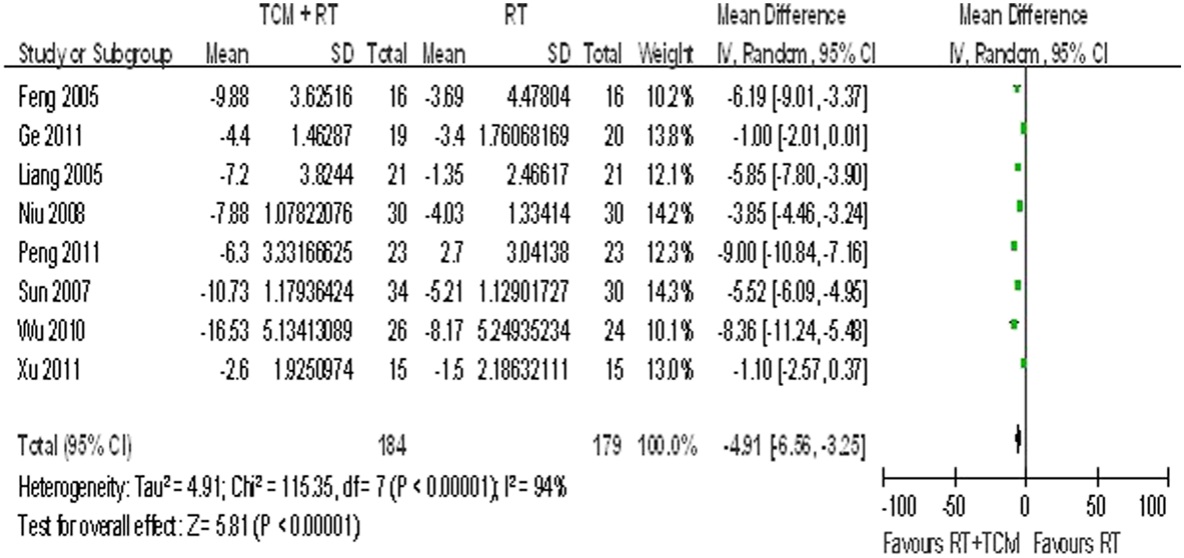
Angina frequency.

Eight studies reported the patients’ angina frequency per week. A random-effect model was applied because obvious heterogeneity was observed among these studies, as seen in Figure 3 (P < 0.00001, I^2^ = 94%). The results indicated that patients in the treatment group experienced angina almost five times less frequently than those in the control group (WMD = −4.91, 95% CI = −6.56 to −3.25).

**Figure 4:**
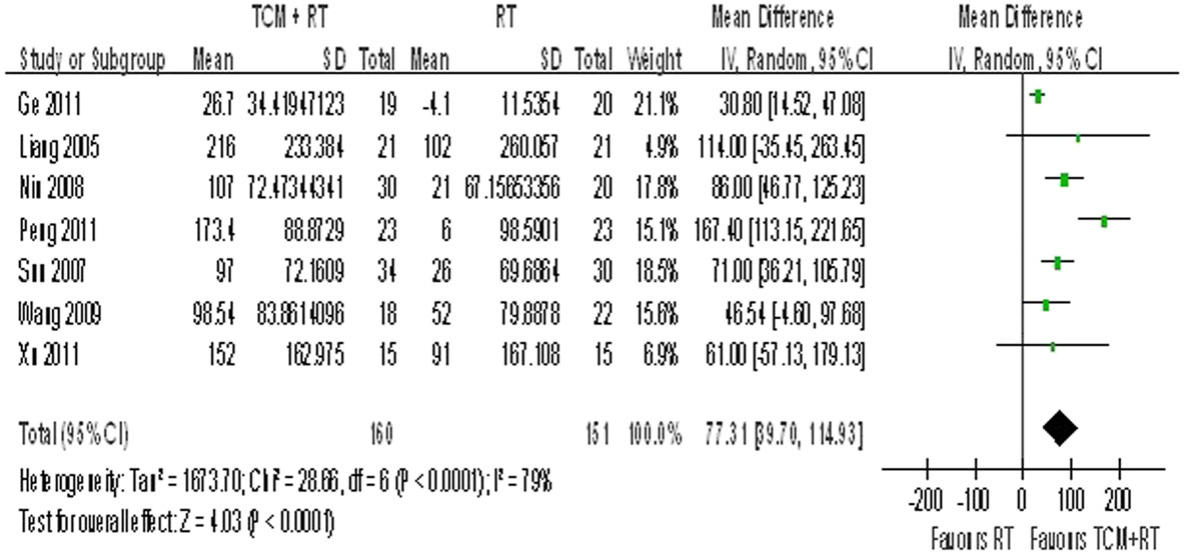
Exercise duration outcome in treadmill test.

Nine studies reported outcomes of the Bruce Protocol Treadmill Test. Either exercise duration or the time to 1-mm ST segment depression was recorded. Exercise duration (measured in seconds) is known to be a health predictor for patients with coronary heart disease [47] and was reported in seven of the nine studies (excluding Wu 2010 and Li 2009). A random-effect model was applied because obvious heterogeneity was observed (P < 0.0001, I^2^ = 79%) (see Figure 4). Patients in the TCM plus routine treatment group had a nearly 1-min improvement in exercise duration (WMD = 77.31, 95% CI = 39.70–114.93).

**Figure 5:**
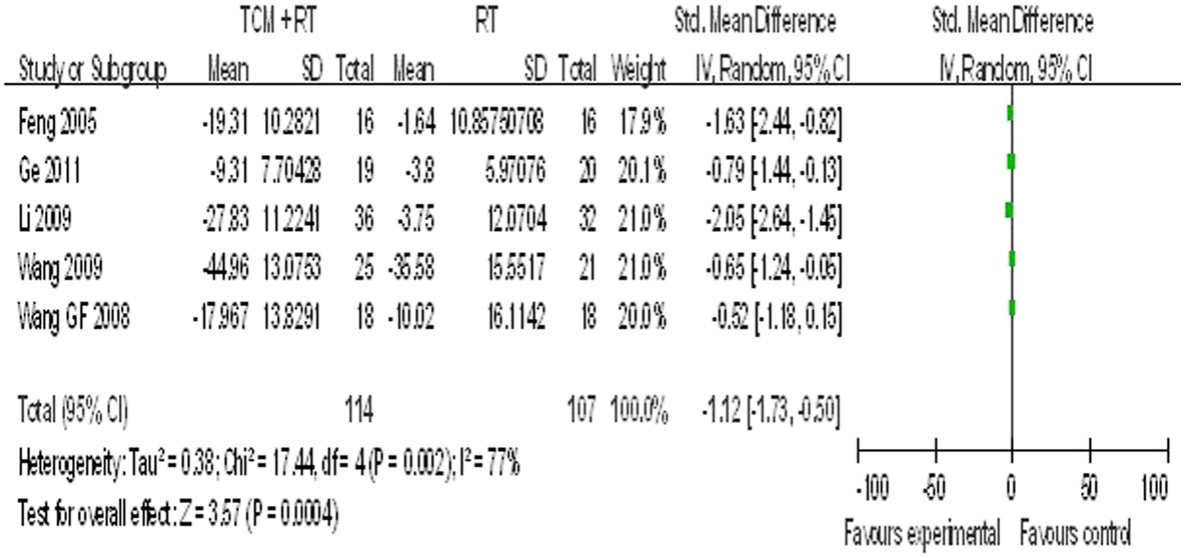
ET-1 (ng/L).

Five studies reported changes in ET-1 levels. As seen in Figure 5, statistical heterogeneity was observed and the units of outcomes varied; thus, a random-effect model was used (P = 0.002, P < 0.05, and I^2^ = 77%). Pooled results indicated greater effects of TCM combined with routine medicine in decreasing ET-1 levels (SMD = −1.12, 95% CI = −1.73 to −0.50).

Spearman correlation analysis using SAS9.1 software showed no correlation between angina improvement and reduced angina frequency or between angina improvement and changes in ET-1 levels.

Group B: TCM vs. RT

**Figure 6:**
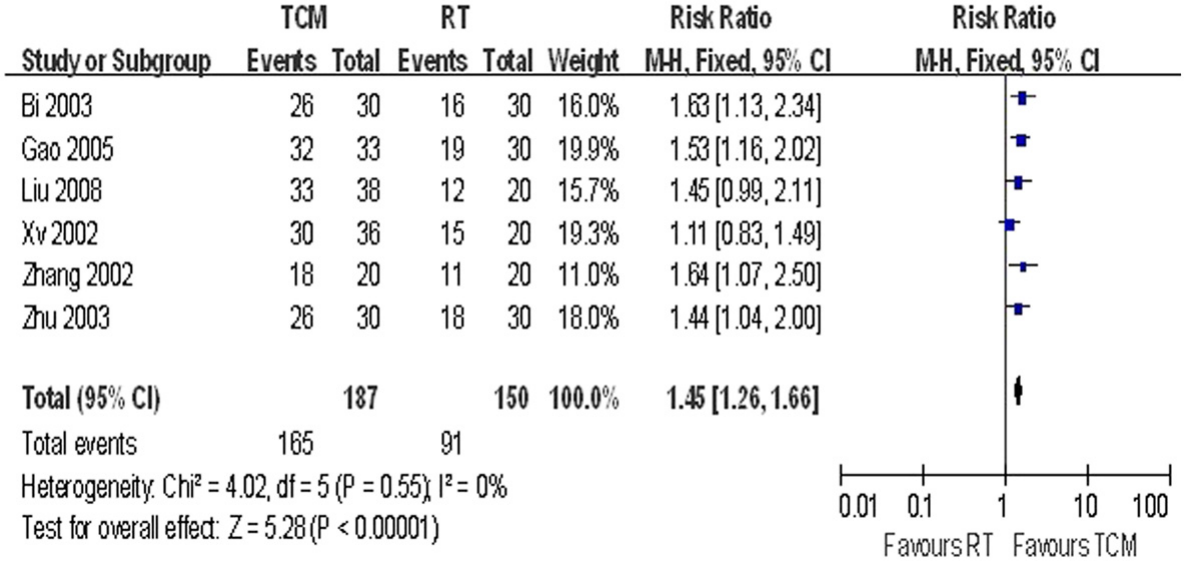
Angina Improvement in Group B.

**Figure 7:**
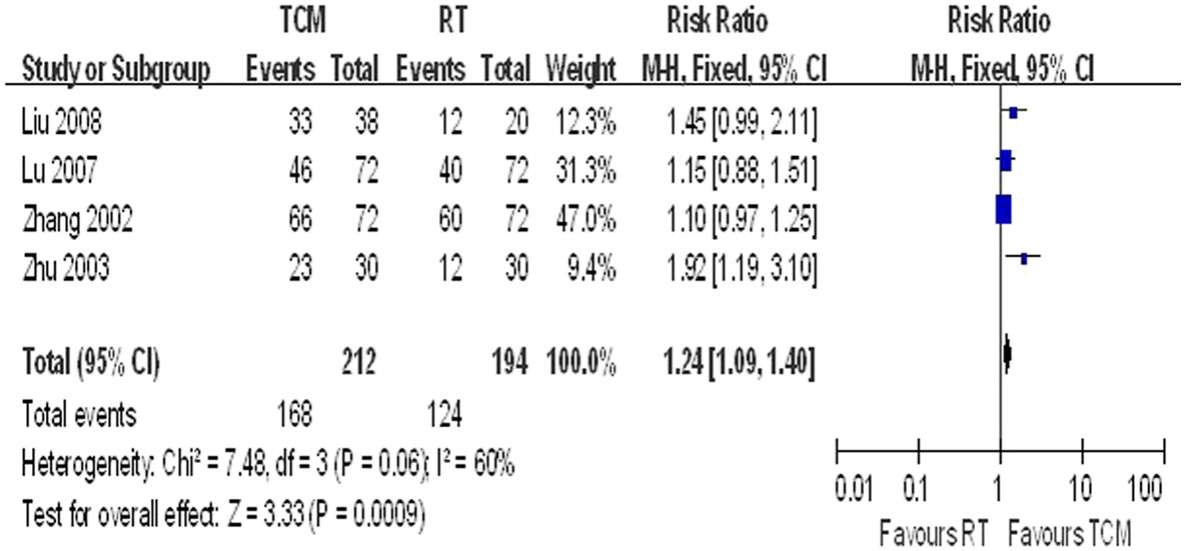
ECG improvement in Group B.

The forest plots in Figures 6 and 7 show no significant heterogeneity across studies (P = 0.55, I^2^ = 0% and P **=** 0.06, I^2^ = 60%, respectively). The pooled results showed OR = 1.45 (95% CI = 1.26–1.66) for angina improvement and OR = 1.24 (95% CI = 1.09–1.40) for ECG tests.

### Sensitivity analysis and meta-regression

No statistical heterogeneity is observed in Figures 2, 6, or 7. However, clinical heterogeneity across trials still needs special attention. The final effect sizes were influenced by multiple factors such as sample size, medication course, publication year, and even different forms of Chinese medicine. As seen in Figures 3 and 4, meta-regression was conducted to test the reliability of the pooled analysis and search for possible causes for this heterogeneity. We conducted meta-regression by residual maximum likelihood (REML) with Knapp-Hartung modification. Tau2 was equal to 0.02809 in Table 3, 6.82 in Table 4, and 1700 in Table 5 as REML estimates of between-study variance. I^2^ was 0.02809%, 91.27%, and 61.44% in terms of the proportion of residual variation due to heterogeneity. The adjusted R^2^ value was equal to −684.96%, 15.63%, and 0.65%, with the proportion of between-study variance explained. Data in Tables 3, 4, and 5 suggest that these four clinical aspects of heterogeneity had no statistically significant effect on the final results. However, only four studies used an identical form and dosage of TCM intervention (*Tongxinluo* capsules), which shows that clinical heterogeneity existed and that its influence cannot be ruled out. According to statistical tests, the results shown in Figure 2 are more likely to reflect the real effects of TCM. Interpretation of the results in Figures 3 and 4 is controversial.

**Table 3:** Meta-regression of basic characteristics of RCTs and ORs of angina improvement

**logRR**	**Coef.**	**Std. Err.**	**t**	**p**	**[95% Conf.**	**Interval]**
Publication year	−0.0362491	−0.53	0.0690226	0.627	-.2278864	0.1553883
Sample size	0.0006184	0.09	0.0072663	0.936	−0.019556	0.0207929
Medication course	0.0027627	0.25	0.0108927	0.812	−0.0274803	0.0330058
Type of intervention	0.12211139	0.60	0.2020443	0.578	−0.4388511	0.683079
_cons	72.78822	0.52	138.6524	0.627	−312.1727	457.7491

**Table 4:** Meta-regressions of basic characteristics of RCTs and mean difference of angina frequency

**Mean difference**	**Coef.**	**Std.err**	**T**	**P**	**[95% Conf.**	**Interval]**
Publication year	8.70691	19.39486	0.45	0.697	−74.74244	92.15626
Sample size	.3612435	3.40234	0.11	0.925	−14.27784	15.00033
Medication course	.3836948	2.185376	0.18	0.877	−9.019221	9.786611
Type of interventions	67.53072	71.69857	0.94	0.446	−240.9633	376.0248
_cons	−17557.2	39125.53	−0.45	0.698	−185900.8	150786.4

**Table 5:** Meta-regressions of basic characteristics of RCTs and mean difference of Treadmill Test results

**Mean difference**	**Coef.**	**t**	**Std. Err.**	**P>t**	**[95% Conf.**	**Interval]**
Publication year	0.1781444	0.4136524	0.43	0.696	−1.138282	1.494571
Sample size	0.0122943	0.1209633	0.10	0.925	−0.3726648	0.3972535
Medication course	−0.0931837	0.0898212	−1.04	0.376	−0.3790348	0.1926673
Type of interventions	−4.132576	2.23568	−1.85	0.162	−11.24751	2.982355
_cons	−352.001	831.7356	−0.42	0.701	−2998.955	2294.953

### Safety analysis and publication bias

**Figure 8:**
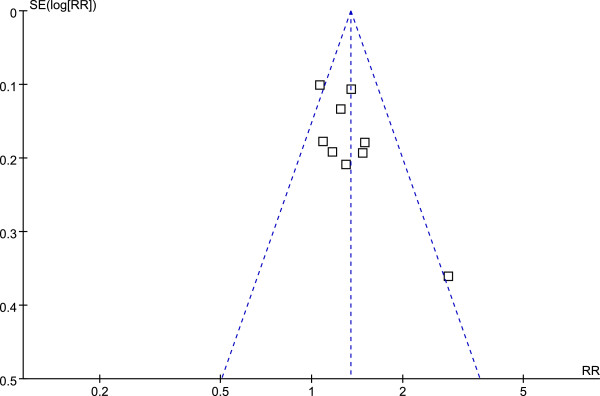
Funnel plot of angina improvement in Group A.

**Figure 9:**
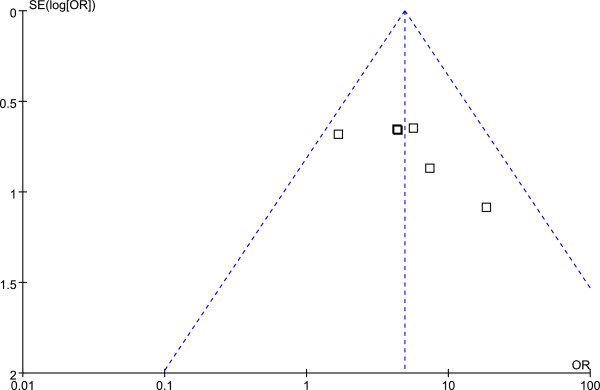
Funnel plot of angina improvement in Group B.

Ten of the 21 RCTs reported routine blood or urine examination results as well as liver and kidney function test results. No side effects were reported with the exception of one study (Li 2009) that reported two cases of stomachache in the treatment group. Funnel plot analysis (Figures 8 and 9) showed no significant publication bias.

## Discussion

### Findings

The results of this study suggest that women are more likely to suffer from CSX based on the fact that patients were randomly recruited.

Improving patient outcomes is the primary goal of CSX management. Compared with routine Western medicine, TCM has the potential of being more effective in relieving symptoms, improving ECG results, and prolonging exercise duration in treadmill tests; however, these advantages were not obvious based on the evidence collected so far. The results in Groups A and B show that Chinese medicine with or without routine Western medicine could improve the degree of angina and reduce the frequency of angina attacks by five times per week. Besides these presenting symptoms of the patients, objective measurements such as ECG and treadmill testing also provided evidence. Because CSX and coronary heart disease share the same symptom of chest pain, the effective solution for the treatment of CSX may provide useful information in terms of symptom improvement for coronary heart disease. Studies in this field will be meaningful.

One mechanism of action of the Chinese herbal medicines prescribed to patients with CSX is believed to involve regulation of endothelial function. A number of cell factors, including ET-1, ET-21, CRP, hs-CRP, and ET-1, were measured and recorded in most of the involved studies. In addition, a comprehensive study (by one researcher, C.J.) of the pharmacological actions of all 48 types of herbs involved in TCM showed that these herbs can improve or protect endothelial function. Analysis of the forest plot also provided hints regarding this hypothesis. However, the undetected association between ET-1 levels and angina improvement in this study may have been caused by the small size of the studies.

Furthermore, we identified a total of 259 case reports and case series from our search, almost 12 times the number of RCTs conducted on this topic. Observational studies have been published since the early 1990s, whereas the first RCT was carried out in 2002. This shows TCM researchers’ growing interest in CSX studies and the difficulties they faced in conducting high-quality RCTs. However, observational studies might also be valuable resources for further research.

Finally, it should be pointed out that the outcomes reported in the included studies in this review were all short-term outcomes, such as symptom relief before and after treatment. The long-term effects of TCM on patients with CSX and its role in the prognosis were scarcely discussed. This may provide a starting point for future studies.

### Limitations

This research only included articles published in English and Chinese. Studies published in other languages were not considered. The sample sizes of the present studies were small, which may lead to bias. The 21 trials included in this review were of moderate to low quality. As a result, the evidence generated needs to be interpreted with caution.

### Implications for further study

These clinical trials show that the basic mechanism of Chinese herbs in relieving chest pain mainly involves endothelial function and the ET-1 pathway. This should draw our attention because Chinese herbs may be helpful for developing and optimal for the treatment of CSX or even coronary heart disease. The methodology of RCTs should be modified in terms of double blinding and allocation concealment. Female patients suffering from CSX should receive more attention.

## Conclusion

This review is the first to systematically evaluate the effects of TCM in the treatment of CSX, addressing the lack of this type of research. In conclusion, TCM shows potential in treating CSX, but its efficacy seems to be minor thus far. Rigorous and multicenter, large-scale clinical trials must be carried out to reveal the exact effectiveness of TCM.

## Competing interests

No competing financial interests exist.

## Authors’ contributions

SHC and MJY developed the idea and designed the research. WJY developed the search strategy, ran the search strategy with CJ, selected which studies to include and extracted data from studies with XL, interpreted the analysis and drafted the final review. MW obtained copies of studies and revised the writing. ZJB carried out the analysis. All authors read and approved the final manuscript.

## Pre-publication history

The pre-publication history for this paper can be accessed here:

http://www.biomedcentral.com/1472-6882/13/62/prepub

## Supplementary Material

Additional file 1 PRISMA 2009 checklist. Click here for file
